# The distal intestinal microbiome of hybrids of Hainan black goats and Saanen goats

**DOI:** 10.1371/journal.pone.0228496

**Published:** 2020-01-30

**Authors:** Shuaiming Jiang, Dongxue Huo, Zhengkai You, Qiannan Peng, Chenchen Ma, Haibo Chang, Xue Lin, Lu Wang, Jiachao Zhang

**Affiliations:** College of Food Science and Engineering, Hainan University, Haikou, Hainan, P. R. China; University of Illinois, UNITED STATES

## Abstract

Intestinal microbiota performed numerous important functions during digestion. The first filial generation (F1) hybrids of Hainan black goats and Saanen goats had different traits, black goats (BG) and white goats (WG), which also brought different production performance. We explored the difference of gut microbiota between black goats and white goats that both belonged to the first filial generation (F1) hybrids. In general, the alpha diversity of the black goat group was significantly higher than the white goat group. The species richness had no significant difference, while the species evenness of BG was higher than WG. *Bacteroides*, *Oscillospira*, *Alistipes*, *Ruminococcus*, *Clostridium* and *Oscillibacter*, as the core gut microbial genera, all had high abundance in BG and WG group. Only the *Bacteroides* and Bacteroidaceae *5-7N15* were the different genera between the BG and WG group, of which *Bacteroides* overlapped with the core genera and enriched in the WG group. Besides, PICRUSt analysis showed that there was a high abundance in the secondary metabolic pathways including membrane transport, replication and repair, carbohydrate metabolism and amino acid metabolism. We found the intestinal microbial species of black goats and white goats were very similar for living in the identical growing environment and feeding conditions, but there was still a slight difference in the content. On the one hand, it was proved that the small effect of genotype and the great effect of diet affected the intestinal microbiota together. On the other hand, it was also proved that these different traits of first filial generation (F1) hybrids may not related to gut microbiota and only because of different genotype. Moreover, characterization of the gut microbiota in BG and WG will be useful in goats gut microbiota research.

## Introduction

Microorganisms are mainly distributed in the oral cavity, alimentary canal, skin epidermis and intestines in animals [1]. There are hundreds of millions microbes in the gut and the total number of intestinal microbial genes is about 100 times more than the total host organism genes [2]. Therefore, the gut microbiota was also known as the second genome of the body [3]. The interactions between microbiota and host including nutrient absorption and immune response, which maintain the stability and host’s health [4]. In some animal species, microbial activity in the cecum may provide 25–35% of animal nutritional needs [5]. Carbohydrates are hydrolyzed and utilized by intestinal microbiota to produce intermediate products, such as monosaccharides, oligosaccharides and organic acids. These intermediates continue to be fermented to produce short-chain fatty acids, mainly including acetic acid, propionic acid and butyric acid [6, 7], which are the main energy source of colon cells, and offer about 10% energy in the food for the host. Besides, proteins are decomposed to produce peptides and amino acids, which are further metabolized by intestinal microbiota to produce short-chain fatty acids [8]. On the contrary, gut microbiota can also use nitrogen sources to synthesize amino acids and proteins as the energy for their growth [9, 10]. For ruminants, different parts of the intestine also have distinct digestive functions. The small intestine is the most vital organ to digest and absorb food and nutrients, including water, inorganic salts, carbohydrate, protein, and fat [11]. The colon and jejunum mainly absorb water and the ileum digests fiber [12]. Large intestine absorbs salt and residual water. The digestion and absorption of food by intestinal microbiota in different individuals is not the same, and it even determines the absorption of nutrients of the host. Turnbaugh *et al*. found that germ-free mice transplanted with intestinal microbiota from obesity mice gained more weight and greater fat than from lean mice [13]. Thus, the difference in the metabolic ability of intestinal microbiota can affect the absorption and utilization of food, which leads to the fat or thin of the host [14, 15].

There are at least 24 indigenous breeds of goats (*Capra hirus*) recorded in China [16]. Hainan black goat, which is peculiar to Hainan province, is a local breed formed by long-term natural selection under the distinct climatic conditions of high temperature and high humidity. It is well known not only for its rough feeding tolerance, strong disease resistance and well adaptability to the tropical maritime climate in Hainan province [17], but also for its delicious meat with no smell of mutton, rich nutrition, and tender meat [18, 19]. Saneng goat is the representative of dairy goat, and Saanen goats have been imported into Hainan province to improve the body size of black goats in the local [20]. The first filial generation (F1) hybrids of Hainan black goats and Saanen goats had different traits, black goats (BG) and white goats (WG), which also brought different production performance including the milk yield was significantly higher in WG, BG gained weight more slowly in the same period. The BG and WG both belong to a first filial generation, while showing different appearance and performance. As we all know, the intestinal microbiota performed numerous activities important during digestion, even influenced phenotype. We supposed these special traits were not only related to their own genetic genes but also related to intestinal microbiota, so we explored the difference of gut microbiota between BG and WG.

With advances in high-throughput sequencing technology, metagenomic sequencing has been used to analyze the diverse species composition of microbiota in different samples [21, 22]. Xu *et al*. revealed the composition and function of cecal microbiota in Dagu chicken using high-throughput sequencing technology in 2016 [23]. The dynamic structure and distribution of small-tail Han sheep microbiota across different intestinal segments also have been studied by high-throughput sequencing technology [3]. As we all know, the gene, environment and proportion of various nutrients in diet affect the stability and balance of intestinal microbiota. In this study, the differences of the gut microbiota and metabolic pathways between the two color types of the hybrids of Hainan black goats and Saanen goats grazing in Hainan province were analyzed by comparing targeted V3-V4 regions using 16S rRNA gene sequencing with the Illumina Miseq platform.

## Materials and methods

### The goats and fecal sampling

The first filial generation (F1) hybrids of Hainan black goats and Saanen goats and were divided into black goats (BG) and white goats (WG), ten each. All the goats were aged from 7–12 months with the body weights ranged from 45 to 67 kg. There was no significant difference in the composition of the feeds which mainly including hay, straw, corn, bran and bean pulp. Faecal samples from 10 black goats (BG) and 10 white goats (WG) were collected and the faecal samples was numbered (BG1-10 or WG1-10). Faecal samples were weighed, mixed with protector (Takara, Japan) in sterile tubes at the ratio of 1:5 (w/w) until homogenous. The mixture was placed in an ice box and transported to the laboratory, and then the metagenomic DNA was extracted immediately. The sampling method and all subsequent methods described in this section were conducted in accordance with the approved guidelines and were approved by the Ethical Committee of the Hainan University (Haikou, China).

### Metagenomic DNA extraction

The commercial metagenomic DNA extraction kit of QIAamp® DNA Stool Mini Kit (Qiagen, Hilden, Germany) coupled with bead-beating was used for DNA extraction from the faecal samples to guarantee the integrity, purity, and concentration of the DNA [24]. The quality of metagenomic DNA was evaluated using 0.8% agarose gel electrophoresis. Metagenomic DNA of the gut microbiota was stored at -20°C prior to further evaluation.

### Amplification V3-V4 regions of 16S rRNA Gene and high-throughput sequencing

The V3-V4 regions of the 16S rRNA genes were amplified using PCR assays [25, 26]. A set of 6-nucleotide barcodes was added to the universal forward primer 338F (5′-ACTCCTACGGGAGGCAGCA-3′) and the reverse primer 806R (5′-GGACTACHVGGGTWTCTAAT-3′), which was targeted at domain bacteria [24]. PCR amplification was achieved following the methods of Wu *et al*. [27]. The PCR products were then purified and quantified using the Agilent DNA 1000 Kit and the Agilent 2100 Bioanalyzer (Agilent Technologies, Palo Alto, USA). Purified PCR products were pooled together in equimolar ratios with a final concentration of 100 nmol/L each and sequenced using an Illumina Miseq PE300 platform.

### Bioinformatics and statistical analysis

Low-quality sequences were removed using the protocols of Zhang *et al*. [28]. After removal of the primers and barcodes, the remaining high-quality sequences were analyzed using the QIIME (v1.7.0) suite of software tools [29]. Approach of phylogenetic investigation of communities by reconstruction of unobserved states (PICRUSt, v1.0) was used to predict the 16S rRNA gene based high-throughput sequencing data for functional features [30]. R program (v3.3.0) was used for statistical analyses. Based on the rarefied OTU subset, the relative abundance of taxa was compared using the Wilcoxon rank-sum test [31]. False discovery rate (FDR) values were estimated using the Benjamini-Yekutieli method to control for multiple testing [32]. PCoA analysis was done in R using the ‘ade4’ package. Major metabolic pathways were visualized with a heatmap made in R using the ‘pheatmap’ package. The sequence data reported in this paper have been deposited in the NCBI database (Metagenomic data: PRJNA347413).

## Results

The gut microbial diversity of 10 black goats (BG) and 10 white goats (WG) was evaluated using 16S rRNA gene high-throughput sequencing. There was a total of 212,177 sequences. From this total 96.48% (204,809 sequences) were high-quality 16S rRNA gene sequences, with an average of 10,240 sequences for each sample (ranging from 8,512 to 11,775). The number of OTUs was analyzed with a confidence coefficient of 97% for each sample ranging from 714 to 1,739, with an average of 1,314 (Table 1). These results indicated that most of the microbial diversity had already been captured.

**Table 1:** Metagenomic sequencing coverage.

Sample	Qualified	OTU Number	Barcode	Observed species
BG1	11195	1608	CCTCTA	2834.97
BG2	11104	1226	TTCACG	2412.93
BG3	11674	1463	ATTCCT	1897.8
BG4	10413	1406	GTCGTA	2479.62
BG5	11045	1663	CCTCTA	2428.05
BG6	9018	1605	CGGATT	1764.45
BG7	10339	1502	GAGTTA	2226.91
BG8	11775	1565	GGTGAA	2194.36
BG9	9057	1283	GGTGCA	1929.41
BG10	10683	1332	GAGTGC	1486.61
WG1	9284	1590	TCTTCA	2509.76
WG2	10254	1739	AGCATC	1882.82
WG3	10333	1144	GTCGGA	1819.93
WG4	10012	1137	AATGTC	2039.96
WG5	11025	1202	TCTACA	1766.72
WG6	9390	1020	AATGGC	1174.14
WG7	8512	714	TTCGCA	1440.54
WG8	9353	845	TTCACA	2066.19
WG9	10698	1142	GATATC	1316.81
WG10	9645	1096	GTCAGA	2407.52

### Alpha diversity and beta diversity between BG and WG

**Fig 1:**
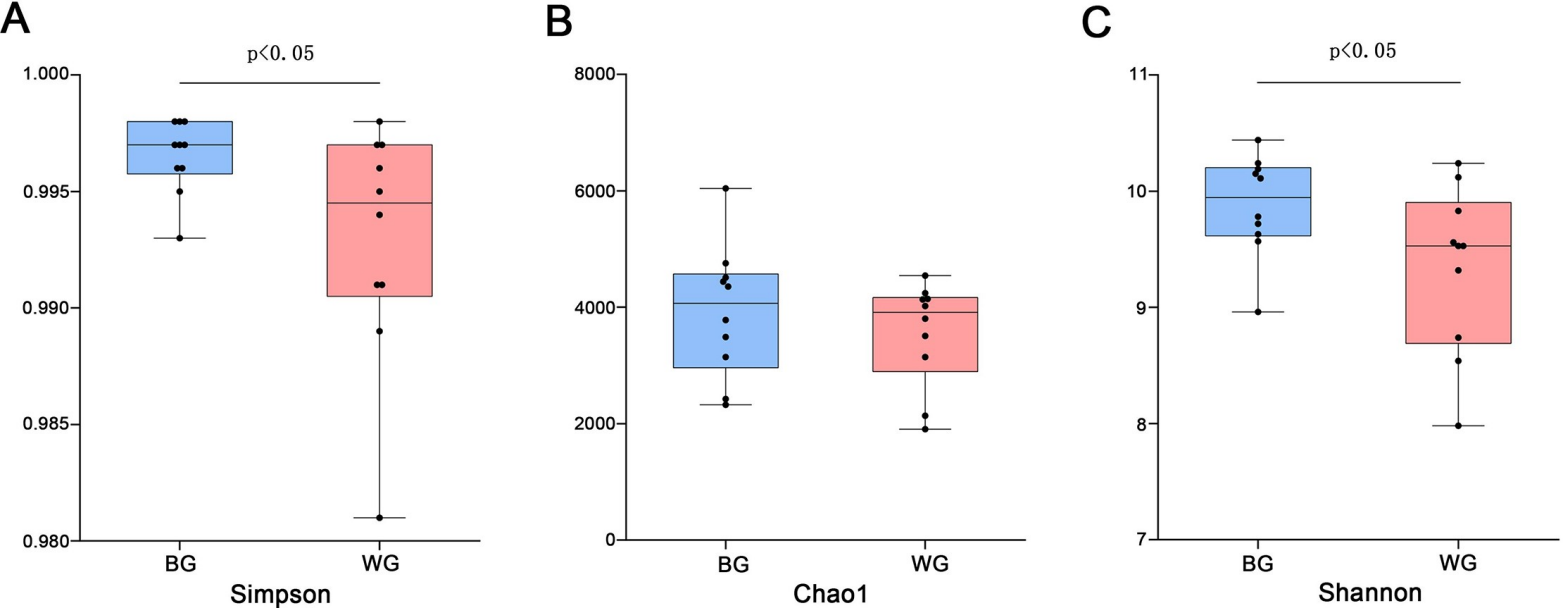
The alpha diversity between black goat (BG) and white goat (WG). P value was calculated using one-tailed unpaired t test.

We evaluated the alpha diversity between BG and WG using Simpson, Chao1 and Shannon index (Fig 1). The Chao1 indices were measured to estimate community richness, which indicated that the species richness had not significantly difference between BG and WG. Simpson and Shannon indexes in the BG were significantly higher than those in WG, which showed the species evenness of BG was higher than WG.

**Fig 2:**
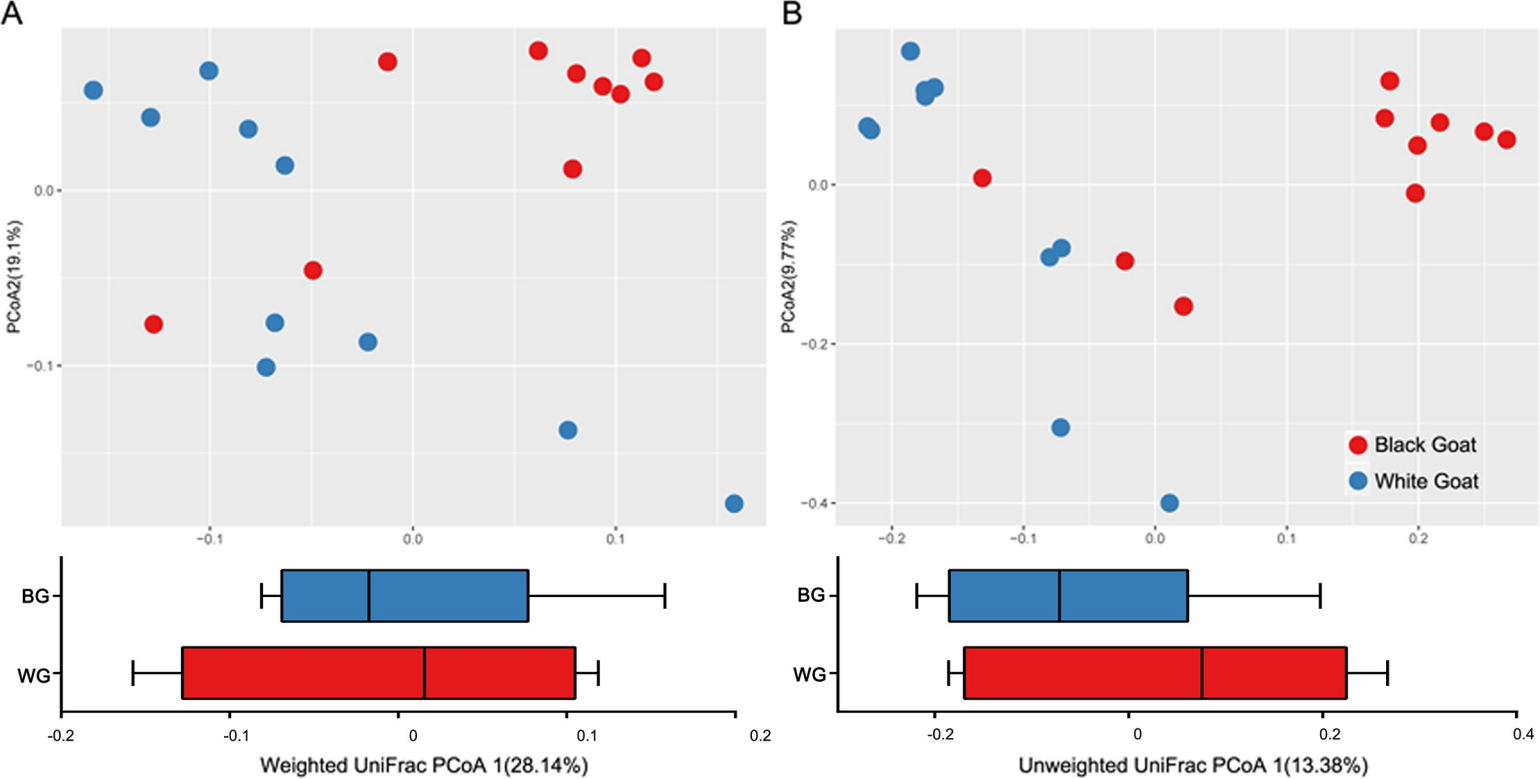
**PCoA score plot based on weighted (A) and unweighted (B) UniFrac metrics for all samples.** Each point represents the composition of the intestinal microbiota of one sample.

Beta diversity was calculated to compare the differences in gut microbiota amongst the faecal samples from BG and WG. Principal co-ordinates analysis (PCoA) was performed based on the weighted and unweighted UniFrac distances of 16S rRNA sequence profiles (Fig 2). This identified an apparent clustering pattern for gut microbiota data from the BG (red) and WG (blue), respectively (Fig 2). Data of BG was clustered in the right side of the coordinate axis, while the data of WG was clustered in the left side of the coordinate axis. There was significant separation between samples from BG and WG (P<0.001), which showed that the structure of gut microbiota was different between BG and WG.

### Core gut microbiota at the level of genus and species

**Fig 3:**
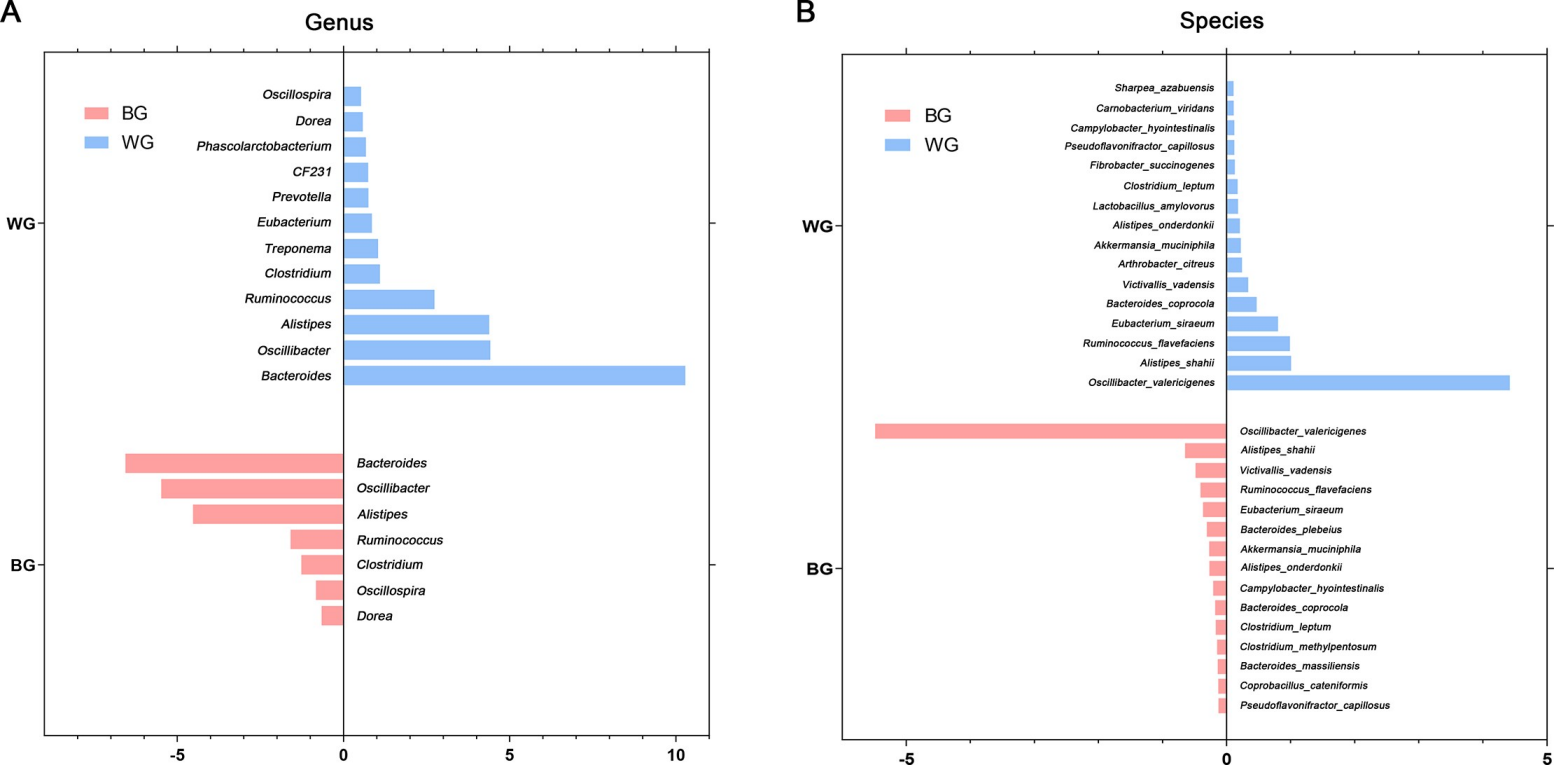
Composition of core gut microbiota of BG and WG at the genus (A) and species (B) level.

**Fig 4:**
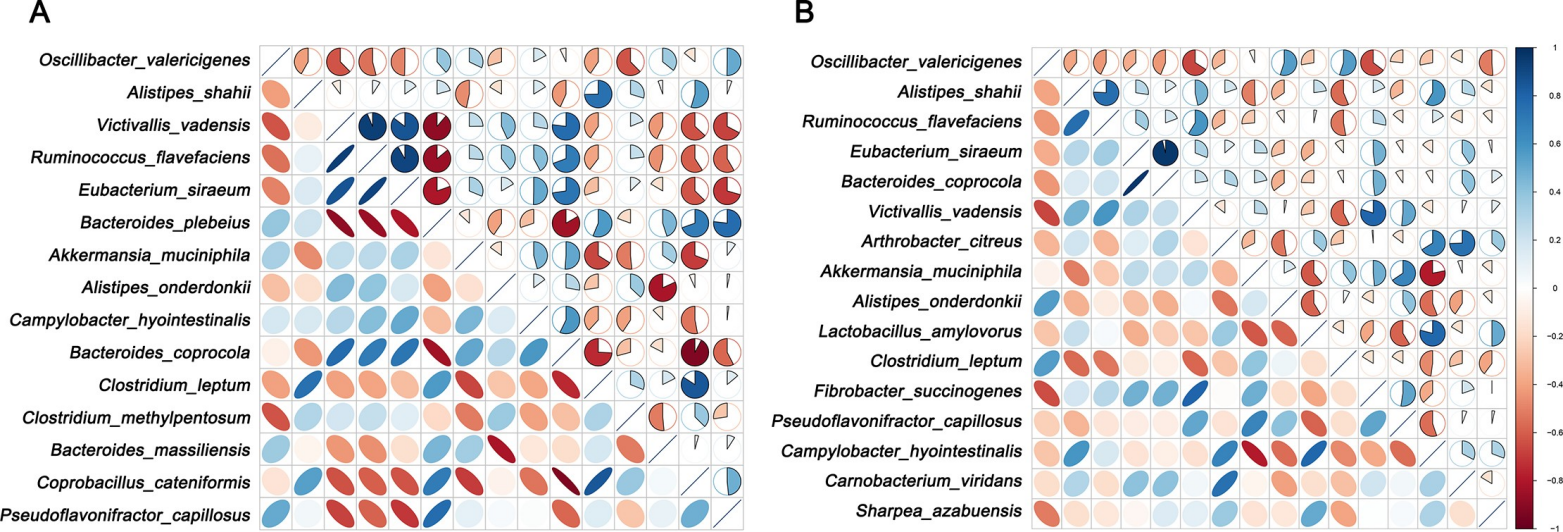
Correlation of core microbiota in BG (A) and WG (B).

The composition of core gut microbiota was identified at the level of genus and species (Fig 3). The microbiota at the genus level that existed in each sample with an average relative content of more than 0.5% was listed in the figure (Fig 3A), and those at the species level were list in Fig 3B. All genera of *Bacteroides*, *Oscillospira*, *Alistipes*, *Ruminococcus*, *Clostridium* and *Oscillibacter* had high abundance in BG and WG group, and *Bacteroides* was the dominant genus. We also found the core gut microbiota had similarity between BG and WG at genus and species level. But interestingly, the dominant species belong to genus *Oscillibacter*, not genus *Bacteroides*. Furthermore, the correlation between core gut microbiota at the species level was analyzed using Spearman's test (Fig 4). Core microbiota of BG was in Fig 4A and WG’s was in Fig 4B. The darker the blue, the positive correlation was stronger. In the same way, the darker the red, the negative correlation was stronger. *Bacteroides_coprocola* had the strong negative correlation with *Coprobacillus_cateniformis* in BG, while had strong positive correlation with *Eubacterium_siraeum*.

### Different genera between BG and WG

The genera with significant differences were listed using the two-tailed t test (Table 2). Only genera of *Bacteroides* and *5-7N15* (belong to Bacteroidaceae family) were the different genera between BG and WG. While at the species level, there was no significant difference between BG and WG. We also found that only *Bacteroides* overlapped with the core gut microbiota and enriched in the WG group.

**Table 2:** The different genera between BG and WG.

Genus taxonomy	p value	BG (%)			WG (%)		
		Median	Min-Max	Average	Median	Min-Max	Average
*Bacteroides*	0.0290	5.850	4.424–9.839	6.566	9.894	5.901–19.430	10.286
Bacteroidaceae *5-7N15*	0.0428	0.044	0.000–0.113	0.045	0.107	0.000–0.276	0.119

### The comparison of major metabolic pathways between samples

**Fig 5:**
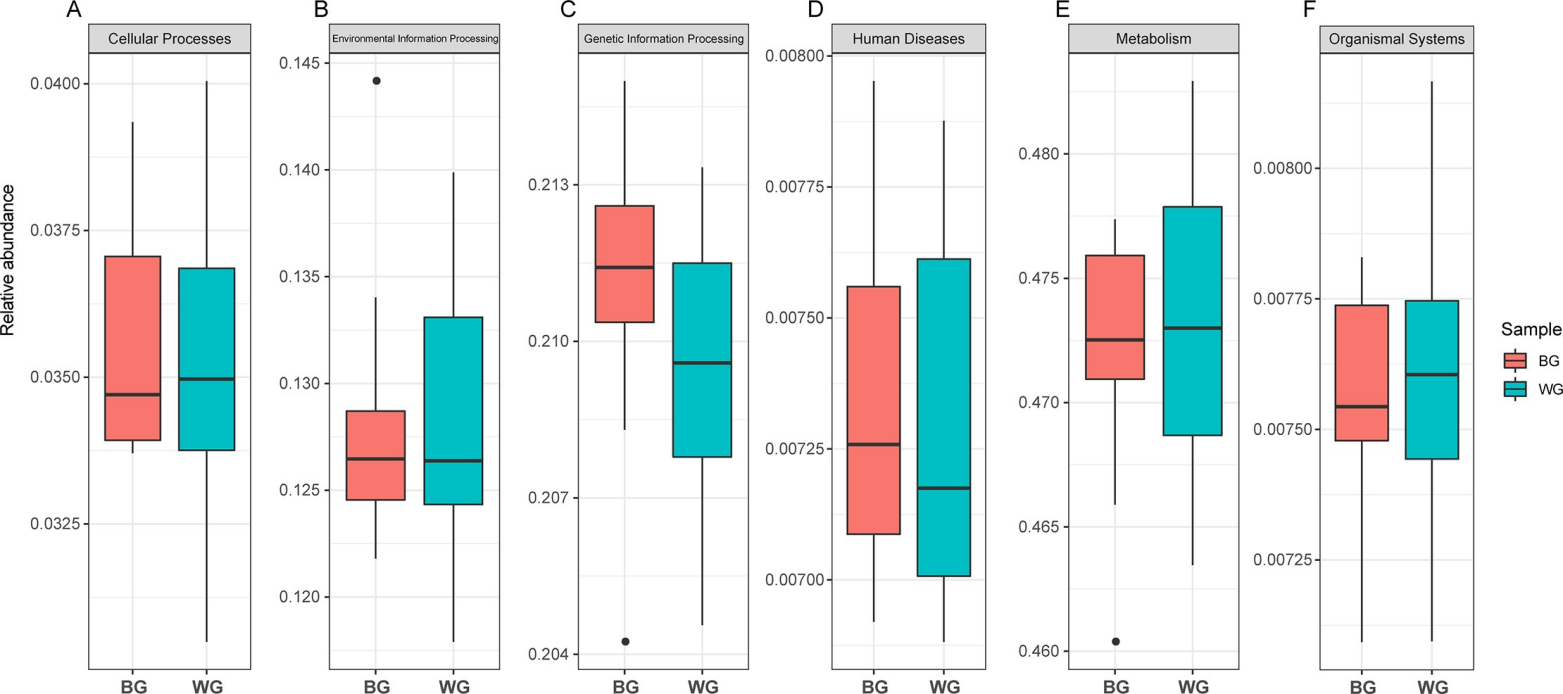
The primary metabolic pathways between BG and WG.

**Fig 6:**
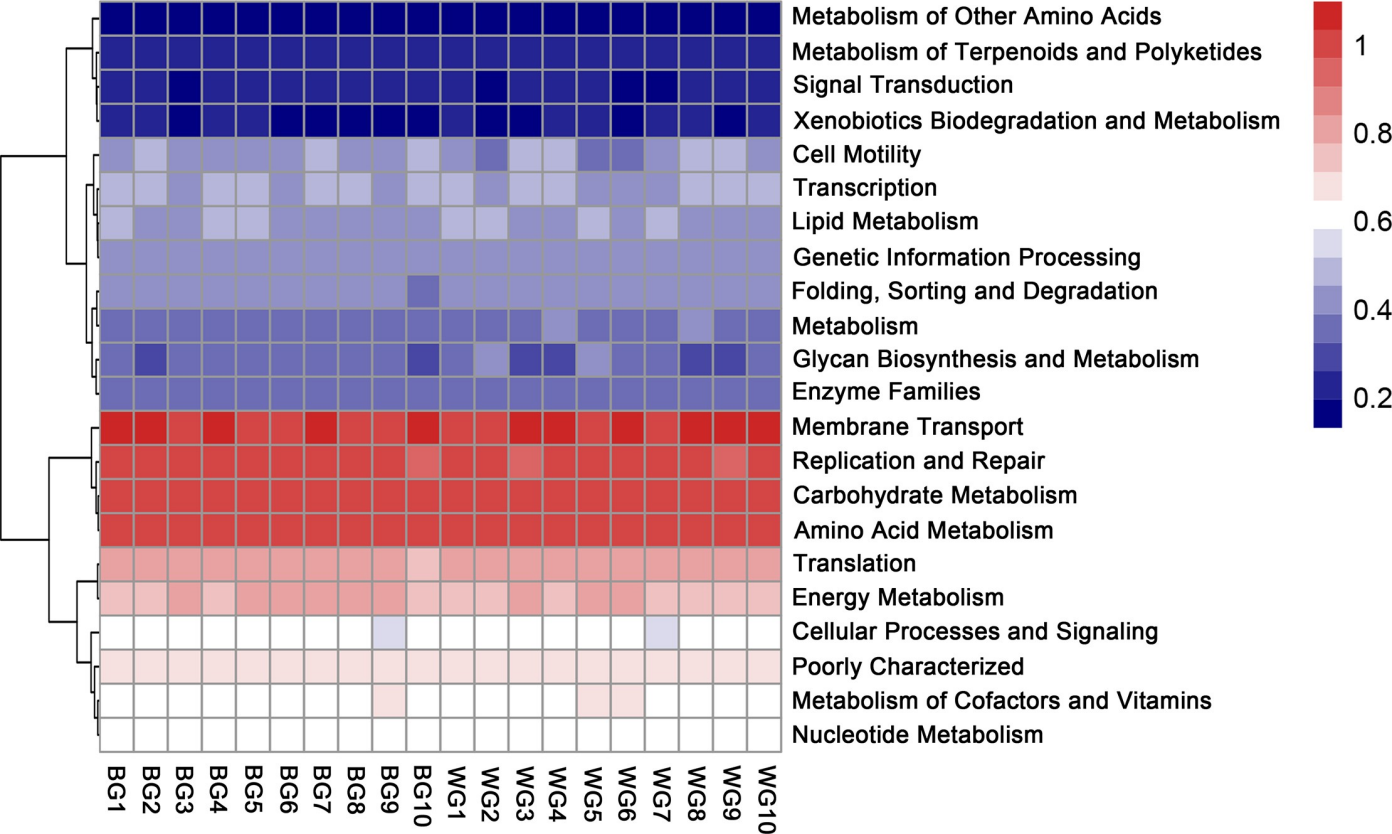
The main secondary metabolic pathways between BG and WG.

PICRUSt (v 1.0) was used to predict functional features based on the V3-V4 regions of 16S rRNA gene sequencing data to determine the potential role of the gut microbiota present in goats. In the primary metabolic pathways, there were no significant differences between BG and WG (Fig 5) and the metabolism pathway had a high abundance above all. The metabolic secondary pathways with relative content greater than 0.01% were listed (Fig 6). Membrane transport, carbohydrate metabolism, replication and repair and amino acid metabolism were the major metabolic secondary pathways and had high abundance in the BG and WG. The two-tailed t test showed that the microbial pathways of metabolism, enzyme families and poorly characterized had significant differences between the BG and WG groups, but due to the low content, it is not obvious in the figure. Furthermore, the tertiary metabolic pathways were predicted, in which pyruvate metabolism pathway had higher abundance in the BG group, to the contrary, peptidases pathway had higher abundance in the WG (S1 Table).

### Correlation between microbiota and microbial pathways

**Fig 7:**
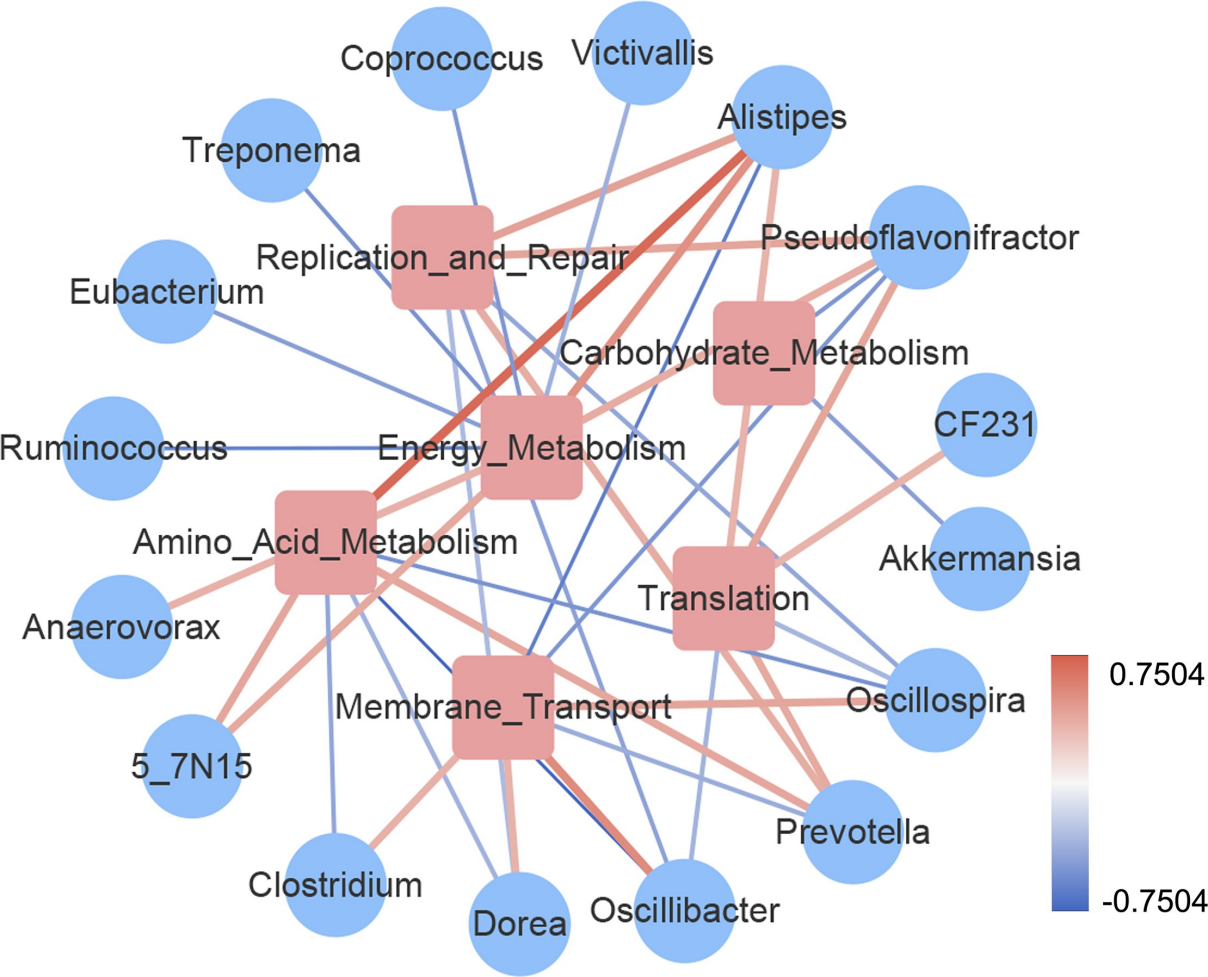
Correlation between genera and metabolic secondary pathways.

We calculated the correlation between the core microbiota and the core metabolic pathways and drew the network (Fig 7), in which the blue circle represented the genera and the pink square represented the core metabolic pathways. If there was a correlation, the genera were connected with the pathways. The thickness of the line indicated the size of the absolute value of the correlation. Additionally, red represented positive correlation, and blue represented negative correlation. We could find that there was a strong correlation between *Alistipes* and amino acid metabolism pathway, indicating that *Alistipes* may play an important role in amino acid metabolism. Bacteroidaceae *5-7N15* was positively correlated with amino acid metabolism and membrane transport.

## Discussion

It used to be believed that the gut was merely an organ to recycle water and some nutrients and store undigested food residues as excrement. In the recent years, sequencing-based assessment of microbial communities in animal faeces had uncovered a large quantity and types of microbial species colonizing in the host gut. A growing body of research had indicated that the microbial communities were associated with the digestion of dietary macronutrients, production of nutrients and vitamins. Thus, the gastrointestinal microbiota can be considered a highly active metabolic organ to complement the host metabolic activities, and the diet can have a major influence on the gut microbial community.

In this research, we used next-generation sequencing of the V3-V4 regions of 16S rRNA genes of metagenomic DNA to understand the full composition of the microbiota in the gut. We compared the alpha and beta diversity between BG and WG and abundant microbiota and major metabolic pathways also had been showed here. Though the goats had different traits, the gut microbiota had high similarity. In general, the alpha diversity of the black goat group was significantly higher than the white goat group. The richness had no significant difference, while the species evenness of BG was higher than WG. *Bacteroides*, *Oscillospira*, *Alistipes*, *Ruminococcus*, *Clostridium* and *Oscillibacter*, as the core gut microbial genus, all had high abundance in BG and WG group. Only the *Bacteroides* and Bacteroidaceae *5-7N15* were the different genera between the BG and WG group, of which *Bacteroides* overlapped with the core genera and enriched in the WG group. Besides, there was a slight difference in the secondary metabolic pathways including metabolism, enzyme families and poorly characterized.

Goats as ruminant animals, numerous microbes habited in the rumen and gut, which were associated with the digestion of dietary macronutrients (including crude protein, non-fibre carbohydrates, non-digestible fibre, and lignin), production of nutrients and vitamins. Studies of the gut microbiota of vegetarian humans found increased levels of *Bacteroides* and *Prevotella* [33]. That revealed that *Bacteroides* had the ability to digest dietary fiber and could explain why both BG and WG had the high abundance of genus *Bacteroides*. High levels of Firmicutes have been reported from the faeces of humans consuming plant-based diets (cereal, vegetables and fruit) where it is thought that they could contribute to the metabolism of the plant-based diet [34]. No matter at the genus level or at the species level, *Oscillibacte*r and *Oscillibacter_valericigenes* both had high abundance. And *Oscillibacter*, *Ruminococcus*, *Eubacterium*, *Clostridium*, *Dorea* and *Oscillospira* all belonged to Firmicutes. Furthermore, an important member of the *Ruminococcus* can metabolize dietary plant polysaccharides including cellulose, xylan and amylase [33].

## Conclusions

This study proved the huge impact of diet on the intestinal tract. We found the intestinal microbial species of black goats and white goats were very similar in the identical environment and feeding conditions, but there was still a slight difference in the content. On the one hand, it proved that the small effect of genotype and the great effect of diet affected the intestinal microbiota together. On the other hand, it also confirmed that these different traits of first filial generation (F1) hybrids may not be related to gut microbiota and only because of different genotype. Moreover, characterization of the gut microbiota in BG and WG will be useful in another gut microbiota-based goat research.

## Supporting information

S1 Table The significant third metabolic pathways between BG and WG. (XLSX)

## Abbreviations

BG: Black goat group
WG: White goat group
PCoA: Principal co-ordinates analysis
16S rRNA: 16 Svedberg ribosomal ribonucleic acid
UniFrac: Unique fraction
OTU: Operational taxonomic unit
DNA: Deoxyribonucleic acid
PCR: Polymerase chain reaction
